# The association of ApoB/ApoA-I ratio with intracranial atherosclerotic stenosis in young patients with ischemic stroke

**DOI:** 10.3389/fneur.2026.1820967

**Published:** 2026-05-21

**Authors:** Meijuan Yan, Lihui Shao

**Affiliations:** 1 Department of Neurology, The Affiliated Municipal Hospital of Xuzhou Medical University, Xuzhou, Jiangsu, China; 2 Department of Rehabilitation, Xinyi Traditional Chinese Medicine Hospital Rehabilitation, Xuzhou, Jiangsu, China

**Keywords:** ApoA-I, ApoB, ApoB/ApoA-I ratio, ICAs, ischemic stroke, young adults

## Abstract

In recent years, the incidence of ischemic stroke among young adults has increased significantly. Identifying risk factors associated with ischemic stroke in this demographic is crucial for effective prevention. This study aimed to investigate the relationship of the serum apolipoprotein B (ApoB)/apolipoprotein A-I (ApoA-I) ratio with intracranial atherosclerotic stenosis (ICAS) in young patients with ischemic stroke. We collected data from patients with ischemic stroke aged 18 to 45 years. ICAS was assessed using computed tomography angiography (CTA), and serum levels of total cholesterol, triglycerides, high-density lipoprotein cholesterol, low-density lipoprotein cholesterol, ApoA-I, and ApoB were measured. The ApoB/ApoA-I ratio was subsequently calculated. Demographic and clinical characteristics were compared between the ICAS group and the non-ICASs group. Multivariate logistic regression analysis was employed to identify independent risk factors for ICAS in young ischemic stroke patients. A total of 161 young ischemic stroke patients were included, with 89 in the ICAS group and 72 in the non-ICAS group. In a univariate analysis, patients with ICAS had a higher prevalence of diabetes mellitus, smoking, and hyperlipidemia, as well as higher levels of ApoB and a higher ApoB/ApoA-I ratio, but lower levels of HDL-C and ApoA-I compared to those without ICAS. After adjustment for age, sex, smoking, hypertension, diabetes mellitus, and HDL-C, multivariable analyses indicated that ICAS had a dose–response relationship with apoB/apoAI ratio quartiles: the second quartile, OR, 3.051 (95% CI, 1.185 to 7.855); the third quartile, 3.516 (1.504 to 8.219); and the fourth quartile, 4.743 (2.109 to 10.667), when referenced to the first quartile. Our study showed that a higher apoB/apoAI ratio is independently associated with ICAS in young stroke patients, after careful exclusion of alternative arteriopathies and adjustment for confounders. However, findings should be confirmed with vessel wall imaging in future studies.

## Introduction

Ischemic stroke remains a leading cause of morbidity and mortality worldwide. Intracranial atherosclerotic stenosis (ICAS) represents a predominant etiology, particularly among Asian populations, although its precise prevalence remains imprecisely defined due to heterogeneity in diagnostic criteria and imaging protocols. Current luminal imaging modalities include CTA, MRA, and DSA (the latter serving as the reference standard), with ≥50% stenosis representing a commonly utilized but variable threshold. A fundamental distinction must be drawn between ICAS as an incidental imaging finding and symptomatic ICAS, in which the stenotic lesion lies within the arterial territory supplying the index stroke—a differentiation critical for accurate etiologic classification and therapeutic decision-making. In Asian-based studies, ICAS has been reported in 30–50% of ischemic stroke cases and approximately one-third of young stroke patients (1–3). Dyslipidemia is a well-established driver of atherosclerosis (4–6), and the ApoB/ApoA-I ratio has been shown to outperform conventional lipid markers in predicting cardiovascular disease (7, 8) and stroke risk in older adults (9). However, its association with symptomatic ICAS in the young stroke population remains unexplored. This study therefore aimed to retrospectively investigate this relationship to identify potential targets for early prevention.

### Study subjects

Between April 2017 and December 2025, we selected patients from a registry of patients consecutively admitted to the Stroke Unit of the Municipal Hospital Affiliated to Xuzhou Medical University. All procedures were approved by the Ethics Committee of the Municipal Hospital Affiliated to Xuzhou Medical University. A total of 161 patients with acute cerebral infarction were enrolled, including 119 males and 42 females, aged 18–45 years. The diagnosis of cerebral infarction complied with the criteria established by the Fourth National Cerebrovascular Diseases Academic Conference and was confirmed by cranial CT or MRI. All patients were admitted within 48 h of symptom onset. The exclusion criteria were as follows: (1) hemorrhagic cerebrovascular disease; (2) trauma; (3) cardiogenic cerebral embolism or cerebral embolism from other causes; (4) acute myocardial infarction; (5) severe hepatic or renal diseases; (6) malignant tumors; (7) intracranial infections; (8) history of familial dyslipidemia; (9) presence of other diseases or factors affecting lipid levels (e.g., hepatic disorders, thyroid diseases, renal diseases, pancreatic diseases, obesity, glycogen storage diseases, gout, Addison’s disease, Cushing’s syndrome, dysglobulinemia, substance abuse, or lipid-lowering medication use prior to onset); (10) those who underwent incomplete vascular imaging and laboratory tests, those who had strokes of other determined etiologies or transient ischemic attacks with negative diffusion-weighted images, and those who did not provide informed consent; (11) non-atherosclerotic arteriopathies.

### Exclusion of non-atherosclerotic arteriopathies

Although only CTA was available for vascular imaging in this retrospective study, we applied the following specific clinical and imaging criteria to exclude non-atherosclerotic causes of intracranial stenosis:

- Moyamoya disease was excluded by the absence of bilateral stenosis or occlusion at the terminal internal carotid artery or proximal anterior/middle cerebral arteries accompanied by characteristic ‘puff of smoke’ collateral vessels on CTA.
- Arterial dissection was excluded by the absence of an intimal flap, double lumen, pseudoaneurysm, or tapered/string-shaped occlusion on CTA, combined with no preceding history of head or neck trauma, neck pain, or Horner’s syndrome.
- Large-vessel vasculitis (e.g., Takayasu arteritis, primary central nervous system vasculitis) was excluded by negative or normal inflammatory markers (erythrocyte sedimentation rate, C-reactive protein, interleukin-6) and the absence of diffuse, long-segment, concentric vessel wall thickening or contrast enhancement on CTA. In cases with clinical suspicion (e.g., unexplained fever, elevated inflammatory markers, or multisystem involvement), additional vessel wall magnetic resonance imaging was recommended; such patients were excluded from final analysis if vasculitis could not be ruled out.
- Reversible cerebral vasoconstriction syndrome (RCVS) was excluded by the absence of multifocal segmental vasoconstriction alternating with dilatation (‘string of beads’ appearance) on CTA and no history of thunderclap headache, acute severe hypertension, or use of vasoactive substances (e.g., cannabis, selective serotonin reuptake inhibitors).
  Patients with any residual suspicion of these alternative arteriopathies after CTA and clinical assessment were excluded from the final analysis. In all patients, the prothrombotic tendency for stroke was evaluated. Data collection.

### Data collection

Based on inclusion and exclusion criteria, after two senior neurologists reached a consensus on the diagnosis, a resident physician or graduate student collected demographic and clinical data of study subjects. Information including age, sex, hypertension, diabetes mellitus, coronary artery disease, smoking status, alcohol consumption, medical history, and usage of antiplatelet and anticoagulant medications was carefully obtained and recorded. Long-term smoking was defined as >10 cigarettes/day for a continuous period of ≥5 years. Long-term alcohol consumption was defined as ≥100 g/day for a continuous period of ≥5 years.

### CTA examination

CTA was performed within 1 week of admission using a Siemens Somatom Definition Flash dual-source CT scanner. Patients were positioned with straps to minimize head movement. The scan range extended from the first cervical vertebra to the vertex. A total of 60–70 mL of iohexol was injected into the antecubital vein at 4 mL/s, followed by a 20 mL saline chaser at 5 mL/s. Employing the Bolus Tracking software, the monitoring plane was positioned at the left common carotid artery bifurcation. Automatic scan initiation was triggered at a threshold of 100 HU with a 2-s delay. Scanning conditions: Tube A 140 kV, 75 mAs; Tube B 80 kV, 318 mAs. Scanning parameters: Collimator width 64 mm × 0.6 mm, pitch 0.7, rotation time 0.5 s. Reconstruction parameters: Slice thickness 1 mm, slice interval 1 mm, reconstruction kernel D30f. After acquisition, data were transferred to Siemens dual-source CT dedicated post-processing software Syngo Via for processing. Two senior radiologists independently assessed image quality and made diagnoses. ICAS was assessed using the method recommended by the Warfarin-Aspirin Symptomatic Intracranial Disease (WASID) trial. The percentage of stenosis was calculated as follows: Stenosis (%) = [1 – (diameter at the stenosis site / diameter of the normal proximal artery)] × 100%. The “normal proximal artery diameter” was defined as the widest, non-tortuous, parallel-walled normal segment proximal to the stenosis. For the intracranial internal carotid artery, the widest normal segment of the petrous portion was used as the reference. In cases where the petrous segment was also diseased, the most distal parallel-walled normal segment was selected. Patients with stenosis >50% or occlusion were classified into the ICAS group, while those with stenosis ≤50% were classified into the non-stenosis group (10). Intracranial arteries included: bilateral internal carotid arteries above the cavernous segment (C3 segment), anterior cerebral artery, middle cerebral artery, posterior cerebral artery, intracranial vertebral artery, and basilar artery. Patients with pure extracranial artery stenosis on CTA were excluded from this study. When concurrent extracranial and ICAS existed, patients were included in the ICAS group. Two independent reviewers, who were blinded to the clinical information, evaluated the vascular images. The *κ* statistics for the concordance rate showed a high degree of agreement (κ = 0.87, *p* < 0.01). Discrepancies were resolved by consensus.

### Definition of symptomatic ICAS

A stenosis was classified as symptomatic ICAS if all of the following criteria were met:

(1) Topographic correspondence: The stenosis was located in the artery directly supplying the acute DWI lesion. Causality was graded as definite (terminal supply zone infarct, no competing mechanism), highly probable (watershed or territorial infarct with stenosis ≥70%), or possible (partial overlap). Only definite/highly probable cases were included in the primary analysis.
(2) Competing mechanism workup: Standardized evaluation included ECG, ≥24-h Holter, echocardiography, and cervical artery imaging.
(3) Handling of coexisting mechanisms: Unlike strict exclusion, we adopted a hierarchical approach. Patients with high-risk cardioembolism (e.g., persistent atrial fibrillation, left ventricular thrombus), dissection, or vasculitis were excluded from symptomatic ICAS. Those with low-to-moderate risk mechanisms (e.g., paroxysmal atrial fibrillation, PFO, small vessel disease) were co-classified as “symptomatic ICAS with possible coexisting mechanism” and included in prespecified sensitivity analyses to assess robustness of findings.

For multi-vessel or tandem lesions, the symptomatic vessel was identified by infarct territory matching and infarct pattern (territorial vs. watershed), with indeterminate cases reported separately.

### Blood lipid tests

Within 48 h of admission, 2 mL of venous blood was collected from the upper limb in a fasting state after an overnight fast for >8 h. The Synchron series biochemical system (Beckman Coulter, USA) was used to measure total cholesterol (TC), triglycerides (TG), low-density lipoprotein cholesterol (LDL-C), and high-density lipoprotein cholesterol (HDL-C). Immunoturbidimetry kits (Shanghai Beijia Biochemical Reagent Co., Ltd.) were used to determine ApoA-I and ApoB levels, and the ApoB/ApoA-I ratio was calculated. Normal reference range: TC: 3.10–5.70 mmol/L, TG: 0.56–1.70 mmol/L, LDL-C: 1.70–3.64 mmol/L, HDL-C: 1.03–2.07 mmol/L, ApoA-I: 1.00–1.60 g/L, ApoB: 0.63–1.14 g/L.

### Statistical analysis

In univariate analysis participants were stratified into ICAS and non-ICAS groups. Continuous variables were tested for normality using the Shapiro–Wilk test. Normally distributed data are presented as mean ± standard deviation and compared using Student’s t-test; non-normally distributed data are presented as median (interquartile range) and compared using the Mann–Whitney U test. Categorical variables are presented as numbers (percentages) and compared using the Chi-square test. Multivariable logistic regression models were performed to identify possible contributing factors for ICAS. The multivariable model included *a priori* defined potential confounders based on clinical knowledge (age, sex, smoking status, hypertension, and diabetes mellitus) as well as variables with *p* < 0.05 on univariate analysis (HDL-C). The ApoB/ApoA-I ratio was entered into the model as quartiles (first quartile as referent). Significance levels were set at *p* < 0.05 for two-tailed tests. All statistical analyses were performed using SPSS version 26.0 (SPSS Inc., Chicago, IL, USA).

## Results

**Figure 1:**
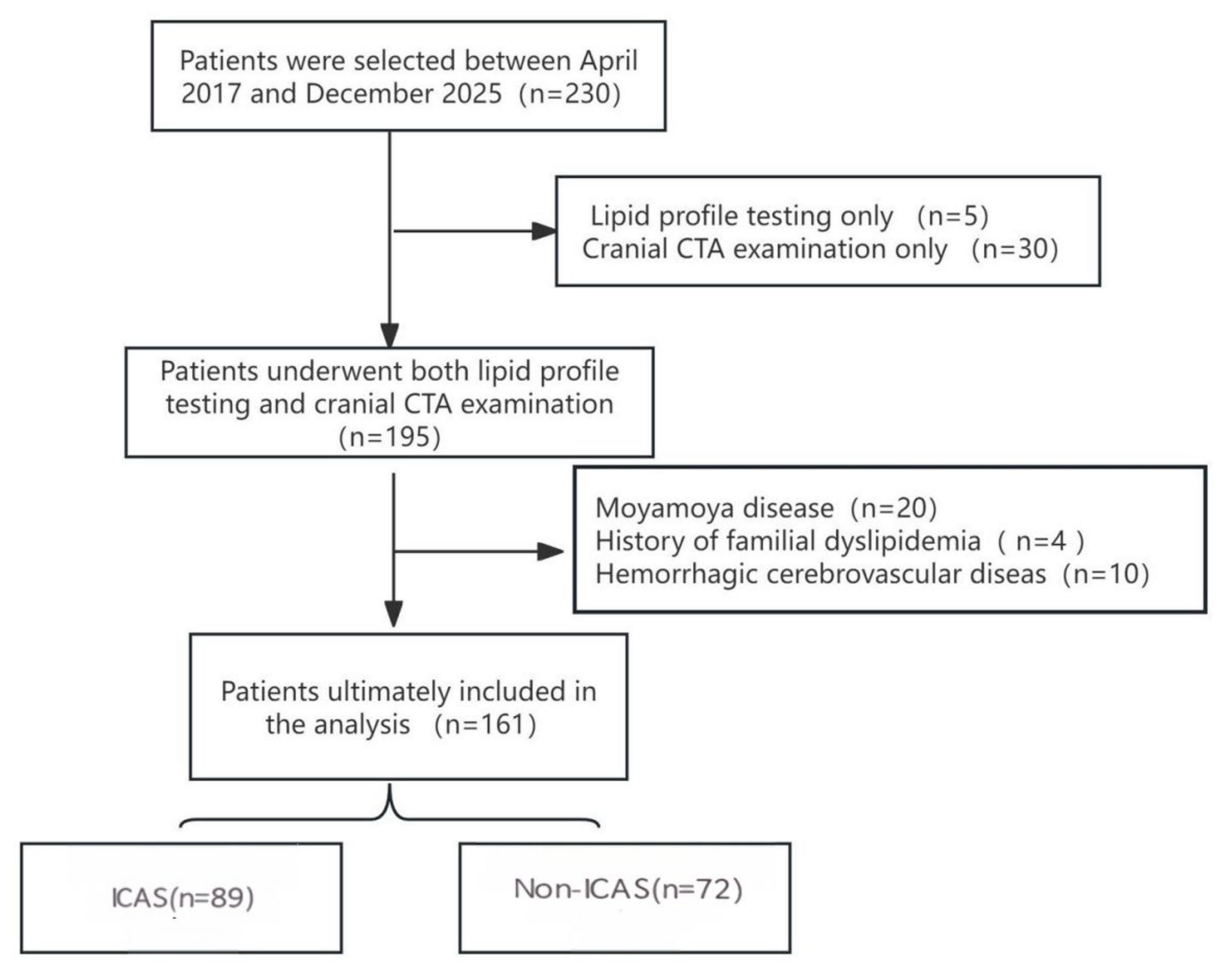
Study participant flow diagram.

A total of 230 young ischemic stroke patients were recruited for analysis, among whom 195 underwent both lipid profile testing and cranial CTA examination. Combining the inclusion and exclusion criteria, 161 patients were ultimately included in the analysis (Figure 1). Among them, there were 119 males and 42 females, aged (39.0 ± 5.7) years. Of these, 89 patients (55.28%) were found to have ICAS, while 72 patients (44.72%) had no ICAS.

The baseline demographic, clinical, and laboratory characteristics of the two groups are listed in Table 1. Compared with patients without ICAS, patients with ICAS had a higher prevalence of diabetes mellitus, smoking, as well as higher levels of ApoB and a higher ApoB/ApoA-I ratio, but lower levels of HDL-C and ApoA-I (all *p* < 0.05). However, there were no significant differences between these two groups in age, sex, the prevalence of hypertension, coronary artery disease, history of stroke or TIA, alcohol consumption, medication use prior to onset, systolic blood pressure, diastolic blood pressure, fasting blood glucose, total cholesterol, triglycerides, and LDL-C.

**Table 1:** Comparison of demographic and clinical characteristics between the ICAS group and the non-ICAS group in young ischemic stroke patients.

Variable	ICAS (*n* = 89)	Non-ICAS (*n* = 72)	*p*
Age (years, mean ± SD)	38 ± 6.41	39 ± 5.11	0.284
Male, *n* (%)	61 (68.54)	58 (80.56)	0.084
HT, *n* (%)	45 (50.56)	27 (37.50)	0.097
DM, *n* (%)	10 (11.26)	2 (2.78)	0.012
CAD, *n* (%)	18 (20.22)	13 (18.06)	0.729
History of Stroke or TIA, *n* (%)	9 (10.11)	7 (9.72)	0.934
Smoking, *n* (%)	39 (43.82)	15 (20.83)	0.002
Alcohol consumption, *n* (%)	22 (24.72)	12 (16.67)	0.213
Medication use prior to onset, *n* (%)			
Anti-platelet agents	12 (13.49)	8 (11.11)	0.650
Antihypertensive agents	20 (22.48)	14 (19.44)	0.902
Anticoagulants	4 (4.49)	2 (2.78)	0.567
SBP (mmHg), mean ± SD	130.31 ± 16.40	127.66 ± 20.15	0.359
DBP (mmHg), mean ± SD	89.67 ± 11.66	88.64 ± 12.68	0.593
FBG (mmol/L), median (IQR)	6.52 (4.54–8.50)	6.49 (4.48–8.50)	0.924
TC (mmol/L), median (IQR)	4.91 (3.79–6.03)	4.63 (3.57–5.69)	0.106
TG (mmol/L), median (IQR)	1.79 (0.89–2.69)	1.55 (0.66–2.44)	0.558
LDL-C (mmol/L), mean ± SD	3.15 ± 0.85	2.98 ± 0.74	0.183
HDL-C (mmol/L), mean ± SD	1.01 ± 0.30	1.16 ± 0.34	0.003
ApoB (g/L), mean ± SD	1.02 ± 0.25	0.89 ± 0.22	0.001
ApoA-I (g/L), mean ± SD	0.89 ± 0.21	1.01 ± 0.26	0.001
ApoB/ApoA-I ratio, mean ± SD	1.14 ± 0.26	0.88 ± 0.21	<0.001

HT, Hypertension; DM, Diabetes mellitus; CAD, coronary artery disease; SBP, systolic blood pressure; DBP, diastolic blood pressure; FBG, fasting blood glucose; TC, total cholesterol; TG, triglycerides; HDL-C, high-density lipoprotein cholesterol; LDL-C, low-density lipoprotein cholesterol; ApoB, apolipoprotein B; ApoA-I, apolipoprotein A-I.

Multivariable logistic regression analysis was performed to identify factors independently associated with ICAS, adjusting for age, sex, smoking, hypertension, diabetes mellitus, and HDL-C. As shown in Table 2, higher ApoB/ApoA-I ratio quartiles were independently associated with ICAS in a dose–response manner. Compared with the lowest quartile (Q1), the adjusted odds ratios for Q2, Q3, and Q4 were 3.051 (95% CI, 1.185–7.855, *p* = 0.021), 3.516 (95% CI, 1.504–8.219, *p* = 0.004), and 4.743 (95% CI, 2.109–10.667, *p* < 0.001), respectively.

**Table 2:** Multivariable logistic regression analysis of factors associated with ICAS.

Variable	Adjusted OR	95% CI	*p*-value
Age	1.192	0.386–4.876	0.873
Male	1.316	0.370–4.675	0.671
Smoking	2.320	0.783–6.875	0.129
Hypertension	1.412	0.115–17.346	0.787
Diabetes mellitus	1.726	0.716–4.158	0.224
HDL-C	0.620	0.053–7.206	0.703
ApoB/ApoA-I ratio quartiles			
First	Referent		
Second	3.051	1.185–7.855	0.021
Third	3.516	1.504–8.219	0.004
Fourth	4.743	2.109–10.667	<0.001

OR adjusted for age, sex, smoking, hypertension, diabetes mellitus, HDL-C, and ApoB/ApoA-I ratio quartiles. HT, Hypertension; DM, Diabetes mellitus; HDL-C, high-density lipoprotein cholesterol; ApoB, apolipoprotein B; ApoA-I, apolipoprotein A-I. * Ranges for sex-specific quartiles were <0.60, 0.60–0.73, 0.74–0.91, and >0.91 for males; <0.62, 0.62–0.73, 0.74–0.87, and >0.87 for females.

## Discussion

The main finding of our study is that a higher apoB/apoAI ratio is independently associated with ICAS in young ischemic stroke patients, and this association remains significant after adjusting for traditional cerebrovascular risk factors. Our results align with recent evidence demonstrating the diagnostic utility of the ApoB/ApoA-I ratio in intracranial atherosclerosis. A study by Li et al. including 408 ischemic stroke patients found that the ApoB/ApoA-I ratio was independently associated with ICAS with a diagnostic AUC of 0.764, sensitivity of 81.3%, and a cutoff value of 0.8122, while no such association was observed for extracranial carotid stenosis (11). Similarly, a study investigating differential distribution of apolipoproteins across ischemic stroke subtypes reported that the ApoB/ApoA-I ratio was significantly higher in ICAS patients compared to those with extracranial atherosclerosis or small artery occlusion, and random forest models identified the ratio as the most influential predictor for stroke subtype stratification (12).

Our study also revealed that LDL-C and TC were not significantly associated with ICAS in young ischemic stroke patients, suggesting that LDL-C and TC may be less sensitive in reflecting the lipid metabolism imbalance associated with ICAS in this population. However, HDL-C was significantly lower in the stenosis group than in the non-stenosis group, with statistical significance. A possible explanation is the higher proportion of smokers and patients with diabetes mellitus in the stenosis group. Jahdkaran et al. found that smoking and diabetes mellitus were closely associated with the occurrence of dyslipidemia (13). Moreover, Wild et al. demonstrated that diabetes mellitus itself could exacerbate dyslipidemia, creating a synergistic effect (14). Notably, Song et al. reported that while cigarette smoking is significantly associated with severe symptomatic extracranial atherosclerosis in older populations, young patients did not show this association and exhibited a relatively higher preference for intracranial involvement (15). Consistent with this, although smoking showed a strong univariable association with ICAS in our cohort (*p* = 0.002), it did not retain statistical significance in the multivariable model after adjustment for the ApoB/ApoA-I ratio and other covariates (OR = 2.320, 95% CI: 0.783–6.875, *p* = 0.129). This attenuation suggests that the effect of smoking on ICAS may be partially mediated by its impact on lipid metabolism, further supporting the concept that apolipoproteins integrate multiple atherogenic pathways.

The prognostic value of the ApoB/ApoA-I ratio in cerebrovascular disease has been increasingly recognized in recent studies. In a prospective cohort study in Taiwan, Chou et al. demonstrated that ApoB level and the ApoB/ApoA-I ratio were significant predictors of ischemic stroke risk, with predictive ability superior to routine clinical lipid measurements (16). Similarly, using data from the China Health and Nutrition Survey (CHNS), Liu et al. found that the ApoB/ApoA-I ratio was positively associated with incident stroke, and this association was stronger than that of the non-HDL-C/HDL-C ratio (17). Beyond stroke risk prediction, the ApoB/ApoA-I ratio has also been implicated in stroke recurrence and functional outcomes. Lin et al. revealed that a high ApoB/ApoA-I ratio was strongly associated with stroke recurrence within 1 year after the first incident stroke, regardless of whether patients had high or low LDL-C levels (18). Furthermore, Li et al. conducted a prospective cohort study of 1,247 patients with acute ischemic stroke and found that a higher ApoB/ApoA-I ratio was independently associated with poor outcomes at 3 months (adjusted OR = 3.986, 95% CI: 2.220–7.155) (19). Collectively, these findings, together with our results demonstrating an independent association between the ApoB/ApoA-I ratio and ICAS in young stroke patients, suggest that this biomarker reflects the underlying burden of atherosclerosis and serves as a robust predictor across the continuum of cerebrovascular disease from atherosclerotic stenosis to clinical events, recurrence, and post-stroke recovery.

While the association between the ApoB/ApoA-I ratio and atherosclerosis is well established in general stroke populations, our study extends these findings to young ischemic stroke patients (aged 18–45 years) in several important ways.

First, young patients typically have a lower burden of traditional cardiovascular risk factors (e.g., hypertension, diabetes, hyperlipidemia) compared with older stroke populations. In this lower-risk context, the independent association of the ApoB/ApoA-I ratio with ICAS (adjusted OR = 4.74 in Table 2) suggests that this biomarker may capture atherogenic risk that is not fully reflected by conventional lipid parameters. Of note, the magnitude of association in our young cohort appears comparable to that reported in general stroke populations (OR ≈ 2–3 in older adults) (16, 17). Sabino et al. previously demonstrated that after adjustment for traditional risk factors including sex, age, smoking, hypertension, hs-CRP, and dyslipidemia, the ApoB/ApoA-I ratio remained independently associated with increased risks of ischemic stroke and peripheral arterial disease in young patients (20).

Second, in young adults with ischemic stroke, the differential diagnosis of intracranial stenosis is broader than in older adults, including non-atherosclerotic arteriopathies such as dissection, vasculitis, and moyamoya disease. A landmark study by Ahn et al. using high-resolution magnetic resonance imaging (HR-MRI) in young patients (≤55 years) with minimal atherosclerotic risk factors found that atherosclerosis was actually an uncommon pathology of middle cerebral artery stenosis, with only 27.4% of patients classified as HR-athero while the remainder had HR-MMD, HR-dissection, or HR-vasculitis (21). This underscores the critical importance of distinguishing true atherosclerotic stenosis from other arteriopathies in young populations. Whether an elevated ApoB/ApoA-I ratio could help distinguish atherosclerotic from non-atherosclerotic stenosis remains speculative based on the current cross-sectional data. Future prospective studies with head-to-head comparison against vessel wall MRI are needed to evaluate any potential adjunctive role of this biomarker.

Third, while the association between the ApoB/ApoA-I ratio and ICAS does not directly inform treatment, it generates the hypothesis that lipid-lowering interventions (e.g., statins) might be relevant in this population. However, because our study did not evaluate any therapeutic intervention, no conclusions about treatment efficacy or target lipid levels can be drawn. Future prospective studies are needed to determine whether the ApoB/ApoA-I ratio predicts response to statin therapy or other lipid-lowering strategies in young ICAS patients. Collectively, while the biological role of the ApoB/ApoA-I ratio in atherosclerosis is not novel, its robust association with ICAS in this young cohort—where alternative etiologies are common and traditional risk factors are less prevalent—suggests that further investigation of this ratio in prospective studies is warranted.

The ApoB/ApoA-I ratio was independently associated with ICAS in this young stroke cohort. The pathophysiological basis for its superiority over TC and LDL-C is not yet fully elucidated. One possible explanation is that ApoA-I, the primary protein component of HDL particles, exhibits anti-atherosclerotic properties. Additionally, ApoA-I inhibits the onset and progression of atherosclerosis through multiple mechanisms, including anti-inflammatory and antioxidant effects, enhancement of NO bioactivity, antithrombotic actions, and endothelial function protection (22–24). ApoB is the main structural protein of very low-density lipoprotein (VLDL), intermediate-density lipoprotein (IDL), and low-density lipoprotein (LDL), with one molecule of ApoB present on each of these atherogenic particles. In predicting atherosclerosis, ApoB reflects the total concentration of these atherogenic particles. ApoB has demonstrated superiority over LDL-C in predicting vascular events, establishing it as a more potent risk biomarker than LDL-C (25). Consequently, the ratio of ApoB/ApoA-I provides a more accurate representation of the balance between risk factors and protective factors in the atherosclerotic process. An elevated ApoB/ApoA-I ratio mirrors an adverse imbalance between proatherogenic and antiatherogenic lipoprotein particles, which may result in enhanced atherosclerotic burden. Compared with other blood lipid indicators, apolipoprotein levels remain relatively stable and are less affected by psychological state, dietary habits, stress, or lifestyle factors (26).

This study has several limitations. First, although we systematically excluded several non-atherosclerotic arteriopathies using clinical and CTA-based criteria, the lack of routine vessel wall magnetic resonance imaging or high-resolution conventional angiography represents a major limitation. In young adults with intracranial stenosis, the pretest probability of alternative arteriopathies (e.g., vasculitis, RCVS, dissection) is substantially higher than in older populations. Therefore, the attribution of stenosis solely to atherosclerosis based on CTA alone should be interpreted with caution. Second, while we defined symptomatic ICAS based on topographic correlation and exclusion of competing stroke mechanisms, we acknowledge that a systematic adjudication protocol for coexisting lesions (e.g., concurrent small-vessel disease, white matter hyperintensities, or silent lacunar infarcts) was not prespecified. This may introduce misclassification bias, particularly in young patients with mixed stroke etiologies. Third, CTA was performed within 1 week of symptom onset in all patients. Although this timing is clinically practical, it may not reliably distinguish a fixed atherosclerotic stenosis from transient acute thrombotic changes. Recanalization or thrombus resolution over time could lead to overestimation of the true degree of fixed stenosis when CTA is performed early. Future studies should consider follow-up vascular imaging (e.g., at 3–6 months) to confirm the persistence of stenosis and more accurately differentiate fixed atherosclerosis from reversible thrombotic changes. Fourth, the single-center, observational, and cross-sectional nature precludes definitive causal inference and may introduce unmeasured confounding. The generalizability of our findings to other populations and ethnicities requires validation in multi-center, prospective cohorts. Fifth, apoB and apoAI were measured in the acute period (within 48 h), but these levels have been reported to be generally stable during the 4 weeks after a stroke (27); therefore, the timing is less likely to affect our findings. Finally, interventional and mechanistic studies are warranted to elucidate the causal pathways and molecular mechanisms linking this ratio to plaque stability and clinical outcomes.

## Conclusion

ICAS might be the most common and important atherosclerotic subtype in the cerebrovascular bed worldwide as well as in Asian populations. However, studies on risk factors specific to ICAS have been limited, and unlike extracranial atherosclerotic stenosis (ECAS), biological markers that could serve as therapeutic targets for ICAS have not been well defined. The current study suggests that the ApoB/ApoA-I ratio is a promising biomarker associated with ICAS in young ischemic stroke patients and sets forth the rationale for larger, prospective, multicenter studies across diverse populations.

## Data Availability

The raw data supporting the conclusions of this article will be made available by the authors, without undue reservation.
